# Prospective Validation of a Weightbearing CT Stability Threshold for Subtle Lisfranc Injuries

**DOI:** 10.1177/10711007251374674

**Published:** 2025-10-31

**Authors:** Magnus Poulsen, Stephan M. Röhrl, Anselm Schulz, Are H. Stødle

**Affiliations:** 1 Division of Orthopaedic Surgery, Oslo University Hospital, Norway; 2 Institute of Clinical Medicine, University of Oslo; 3 Division of Radiology, Oslo University Hospital, Norway

**Keywords:** Lisfranc, Lisfranc diagnostics, weightbearing CT, subtle Lisfranc injury, tarsometatarsal instability, prospective study

## Abstract

**Background:**

Accurate evaluation can be challenging in subtle Lisfranc injuries. Although weightbearing computed tomography (WBCT) provides 3-dimensional assessment under physiological load, its role in diagnosing subtle Lisfranc instability and guiding treatment thresholds remains undefined. Currently there is no consensus on the optimal timing, measurement protocol, or stability threshold with WBCT.

**Methods:**

We prospectively recruited patients with nondisplaced (<2 mm), intra-articular fractures and/or avulsion fractures in the tarsometatarsal 1-3 area. To assess Lisfranc joint stability, patients underwent bilateral, single-leg WBCT scans. Medial cuneiform (C1) and second metatarsal (M2) measurements, evaluating the integrity of both the dorsal and interosseous Lisfranc ligament, were combined and compared to the contralateral, healthy side to create a difference score. Threshold of instability was defined as >3 mm C1-M2 difference score. Measurements were tested for agreement using the intraclass correlation coefficient (ICC).

**Results:**

38 patients were included in the study and were able to fully weightbear after a median of 9 days postinjury. Eight patients (21%) had a C1-M2 difference score >3 mm (95% CI, 3.8-5.8) while loading. Instability was confirmed in all 8 patients during a fluoroscopic stress test. The remaining 30 patients, with a C1-M2 difference score <3 mm (95% CI, 0.6-1.1), were classified as stable and received conservative treatment. These patients were followed up with an additional WBCT scan after 12 weeks, and none had signs of Lisfranc instability with a median different score 0.6 mm (95% CI, 0.4-0.7). Our measurement method demonstrated excellent interrater (ICC 0.95, 95% CI, 0.93-0.96) and intrarater agreement (ICC 0.97, 95% CI, 0.96-0.98).

**Conclusion:**

For subtle Lisfranc injuries, adequate WBCT can be performed 9 days postinjury. In our study population, using our dual-measuring method, a C1-M2 difference >3 mm on WBCT reliably identified subtle Lisfranc instability.

## Graphical abstract

**Graphical Abstract:**
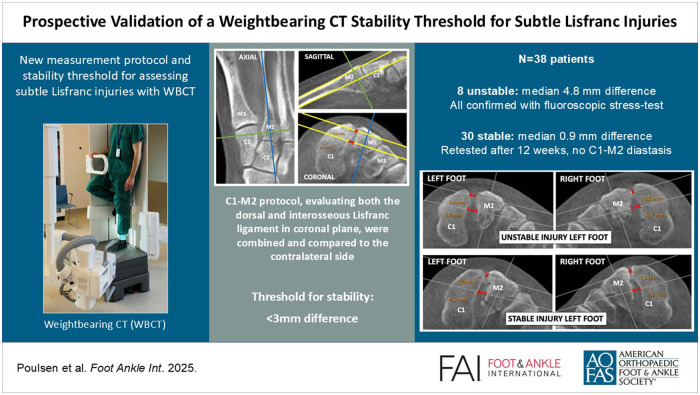
This is a visual representation of the abstract.

**Level of Evidence**: Level II, prospective cohort study.

## Introduction

Damage to the tarsometatarsal (TMT) articulations, commonly denoted as Lisfranc injuries, vary depending on energy and anatomical structures involved.^9,19^ High-energy accidents typically result in extensive fractures and soft-tissue damage whereas low-energy traumas are often characterized by minimal structural damage and can easily go undetected on conventional radiography.^6,17,20^ The latter constitute the most common mechanism of injury, accounting for more than two-thirds of all cases, and are often referred to as subtle Lisfranc injuries.^5,7,27^ Early detection and accurate assessment are crucial for implementing appropriate treatment strategies and minimizing the risk of complications such as progressive midfoot deformity, post-traumatic arthritis, and chronic pain.^9,27^ Therefore, computed tomography (CT) is recommended in the initial evaluation of Lisfranc injuries to identify possible avulsion fractures and subtle joint misalignments.^9^ If the CT examination is negative but there is still a high suspicion of a Lisfranc injury, an MRI scan can be performed.^15^

Once identified, the next step is to assess Lisfranc joint stability, as unstable injuries typically require surgical fixation, whereas stable injuries can effectively be treated with conservative measures.^9,15^ In this assessment, the integrity of the Lisfranc ligaments complex under natural loading is recommended and is best evaluated by comparing the first cuneiform (C1)–second metatarsal (M2) diastasis with that of the healthy contralateral side.^10,26^ Until recently, this assessment was primarily conducted using bilateral weightbearing radiography, with current consensus indicating that a C1-M2 joint space widening greater than 2 mm suggests Lisfranc instability.^9,15,28^ Nevertheless, because of the limitations of plain radiography, this evaluation technique has certain shortcomings in sensitivity. In recent years, weightbearing CT (WBCT) has emerged as a valuable tool for diagnosing complex foot and ankle conditions.^8,11,15^ Its ability to visualize 3-dimensional anatomy and detect dynamic instability positions it as a vital instrument to enhance our clinical decision making. However, existing studies on its application for Lisfranc diagnostics are primarily based on cadaver models or retrospective analyses.^3,4,12,23,25,29^ Volumetric, area, and distance assessments have been introduced; however, there is currently no consensus on the optimal protocol for evaluating these injuries using WBCT.^15^ Only through a clinically tested, standardized measuring technique applied to patients presenting with an acute Lisfranc injury can a given C1-M2 threshold gain clinical significance. Additionally, the optimal time frame for conducting these measurements has yet to be addressed in the literature.

The primary aim of this study was to validate a clinically tailored protocol for measuring Lisfranc joint diastasis using WBCT, to identify the optimal timing following acute injury, and to establish a reliable stability threshold for the Lisfranc joint using this dynamic diagnostic tool.

## Methods

### Inclusion Criteria

A prospective study was initiated to evaluate the stability of the Lisfranc ligament complex following an acute midfoot injury. Inclusion period spanned from April 1, 2023, to May 1, 2024. Although not universally defined in the literature, we considered a subtle Lisfranc injury to be a verified radiologic sign of injury to 1 or more parts of the Lisfranc ligament complex.^5,13,18^ Consequently, all patients with a clinical suspicion of a Lisfranc injury (midfoot pain, swelling, plantar ecchymosis, and/or inability to weightbear) were examined using nonweightbearing radiography and CT of the injured foot. The following inclusion criteria were used based on the CT findings:

1. Nondisplaced (<2 mm) intraarticular and/or avulsion fracture affecting TMT 1, 2, and/or 3.
2. No/minimally (<2 mm) incongruency in the TMT 1-5 joint line.

Additional injuries to the lateral column were not an exclusion criterion.

Exclusion criteria included feet with other significant foot injuries, prior surgeries or injury sequelae involving the TMT joints, open or bilateral injuries, and injuries older than 4 weeks. Only consent-competent individuals aged 18-65 years were enrolled, and all participants signed an informed consent form prior to participation.

### WBCT Setup

**Figure 1.**
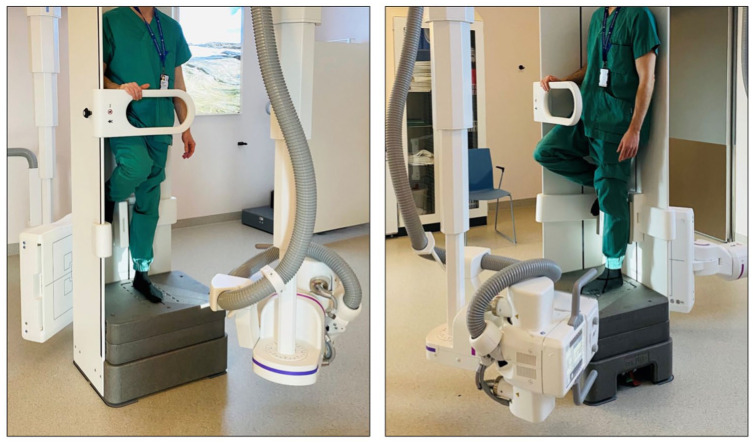
The single-leg stance, weightbearing computed tomography examination was conducted using a twin-robotic X-ray scanner (RAX; Siemens). An identical setup was used for both the injured and healthy foot.

Following enrolment, patients were fitted with a prefabricated walking boot (Swereco; NovaWalk, Kista, Sweden) and instructed to apply light weightbearing on the injured foot as tolerated. A follow-up evaluation was scheduled within 5-7 days to assess their weightbearing capabilities, during which they performed a pain tolerance test by standing on the injured foot for about 10 seconds. If successful, further analysis was conducted within 1-3 days using bilateral, single-leg WBCT with a twin-robotic X-ray scanner (Multitom Rax; Siemens Healthineers, Forchheim, Germany). Full body weight was applied to the examined foot while the contralateral foot was elevated, with the hip and knee flexed at 90 degrees (Figure 1).

### Measurement Protocol

**Figure 2.**
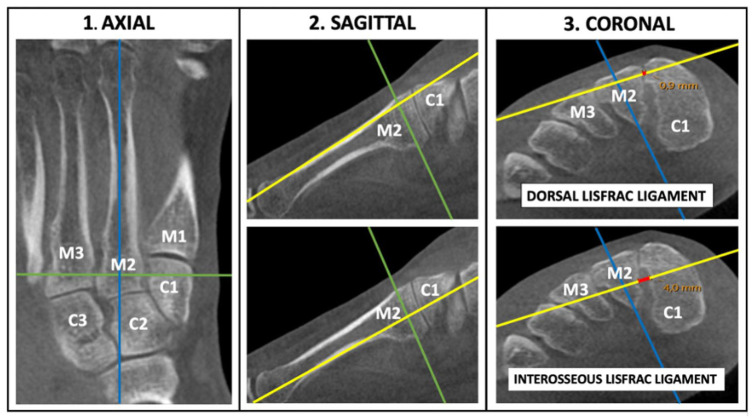
Axes of reference when measuring the dorsal Lisfranc ligament (DLL) and interosseous Lisfranc ligament (ILL) diastasis using bony anatomy. (1) Starting in the axial plane, the appropriate image that visualize the complete M2 bone was used. The sagittal reference line (blue) was placed central and parallel to the M2 shaft while the coronal line (green) was positioned centrally in-between the C1-M2 junction. (2) Second, in the sagittal plane, the axial line (yellow) was placed parallel along the dorsal shaft of the M2 to measure the DLL, or parallel to the plantar shaft to measure the ILL. (3) Finally, in the coronal plane, the sagittal axis (blue) was oriented toward the plantar pole of M2, and the distance between C1 and M2 (depicted as red) was measured along the axial line (yellow). Notably, the intersecting axes are centrally positioned within M2 when measuring the C1-M2 distance. For this patient, the DLL measured 0.9 mm and the ILL measured 4.0 mm. Abbreviations: C1, C2, C3 = medial, middle, and lateral cuneiform; M1, M2, M3 = first, second, and third metatarsal. [See online article for color figure.]

Images were visualized using SECTRA (Linköping, Sweden) IDS 7 software (version 25.2), using the Multiplanar Reconstruction (MPR) feature for analysis. WBCT scans were reconstructed with 0.5 mm thickness. The joint space between the medial cuneiform and the second metatarsal (C1-M2) was assessed using bony landmarks and 1-dimensional measurements. To ensure consistent radiologic metrics, a stepwise, triplane technique was developed and tested for consistency before the study enrollment (Figure 2).

### CT Interpretation, Stability Threshold, and Patient Follow-up

Measurements were conducted continuously for each patient by 2 independent foot and ankle surgeons. For each foot, we used the developed protocol to measure the dorsal Lisfranc ligament (DLL) and the interosseous Lisfranc ligament (ILL) distance, expressed in millimeters. The rationale for evaluating 2 parameters per foot was to enhance the sensitivity of our measurements, provide cross-validation for our findings, and ultimately increase diagnostic accuracy. By combining 2 parameters, we achieved a two-dimensional area evaluation rather than relying solely on a one-dimensional distance assumption.

**Figure 3.**
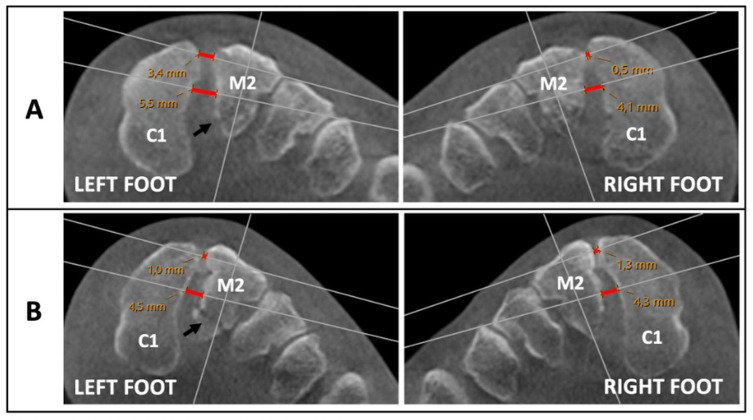
Weightbearing computed tomography assessment in 2 patients (A and B) with a similar-looking, subtle Lisfranc injuries to the left foot. Fracture is indicated with a black arrow. The corresponding measurements of the dorsal Lisfranc ligament (DLL) and the interosseous Lisfranc ligament (ILL) are highlighted in red along the measurement axis. Patient A was classified as unstable, with a difference score of 4.3 mm (left: DLL 3.4 mm, ILL 5.5 mm; right: DLL 0.5 mm, ILL 4.1 mm). Notably, the side-by-side difference of the ILL was only 1.4 mm. Patient B was classified as stable, with a difference score of 0.1 mm (Left: DLL 1.0 mm, ILL 4.5 mm; Right: DLL 1.3 mm, ILL 4.3 mm). C1 = medial cuneiform, M2 = second metatarsal. [See online article for color figure.]

To determine Lisfranc stability, we established a prespecified threshold based on a cadaveric model described by Sripanich et al.^23^ They used WBCT and found a 1.5-mm increase in the ILL distance following complete dissection of the Lisfranc ligament. We applied this criterion to both our 2 parameters, specifically the ILL and the DLL, to establish a combined threshold of 3.0 mm. Consequently, any injury assessment >3 mm was considered unstable (Figure 3). This threshold was expressed as a difference score and calculated using the formula:

$$\begin{array}{l} Difference\;score\;=\;Injured\;foot\;\left(DLL\;+\;ILL\right)\;\\ \;\;\;\;\;\;\;\;\;\;\;\;\;\;\;\;\;\;\;\;\;\;\;\;\;\;\;\;\;\;\;\;\;\;\;\;-Healthy\;foot\;\left(DLL\;+\;ILL\right) \end{array}$$

**Figure 4.**
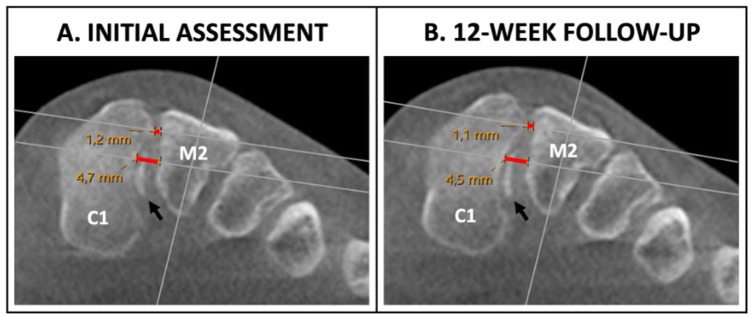
(A) Initial weightbearing computed tomography assessment of a left-sided Lisfranc injury with corresponding measurements of the dorsal Lisfranc ligament (DLL) and interosseous Lisfranc ligament (ILL) distance noted in red (DLL 1.2 mm, ILL 4.7 mm). Notice the presence of an avulsion fracture (black arrow) originating from the ILL inside the C1-M2 junction. After comparing results with the healthy foot, the injury was determined as stable. (B) Twelve-week follow-up evaluation showing preserved C1-M2 stability (DLL 1.1 mm, ILL 4.5 mm). C1 = medial cuneiform, M2 = second metatarsal. [See online article for color figure.]

In cases where an avulsion fracture was present in the C1-M2 joint space, measurements were taken from the originating cortical bone (Figure 4).

Unstable injuries were tested perioperatively with a forefoot abduction stress-test. For verification of instability, the following criteria were necessary: (1) malalignment >2 mm between the medial border of M2 and C2, and (2) C1-M2 distance >5 mm. If present, patients were treated with open reduction and internal fixation using minimally invasive surgical techniques. Conversely, injuries deemed stable were treated conservatively with a walking boot for additional 6 weeks, allowing weightbearing as tolerated in this period. Subsequently, patients were advised to continue using the walking boot as necessary for an additional 6 weeks before undergoing a follow-up assessment with bilateral WBCT after 12 weeks. On completion of the study, a designated radiologist reassessed all WBCT images using the same measurement protocol to further evaluate interobserver agreement.

### Statistical Analysis

Based on the exploratory study design, sample size and power calculations were not performed before study start. All measurements were tested for inter- and intrarater agreement using the intraclass correlation coefficient (ICC) with a 2-way random effects model. Interpretation of ICC results followed the given values: 0-0.1 none, 0.11-0.4 slight, 0.41-0.6 fair, 0.61-0.8 moderate, and 0.81-1.0 substantial.^21^ Numeric data are presented by the median, along with its corresponding range and standard deviation (SD). Additionally, the difference scores are provided with their median, accompanied by a 95% CI. The optimal diagnostic threshold was determined retrospectively using the Youden index approach, which maximizes the sum of sensitivity and specificity minus 1, providing the best numeric balance between sensitivity and specificity. CIs for this threshold were derived by the bootstrap method with 1000 repetitions. For our stable injuries, we compared different score between initial assessment and follow-up evaluation using a paired *t* test based on the assumption that there would be no change in mean difference score. Significance was evaluated at *P* *<.*05. All statistical data analyses were performed using Stata (version 18.5; StataCorp, Texas, USA).

## Results

During the inclusion period, 38 patients were enrolled in the study (Table 1). All patients had CT-verified injuries related to the middle column of the midfoot (Table 2). Discounting 2 patients who were diagnosed late (20 and 21 days postinjury, respectively), all patients were able to adequately load the injured foot within a median of 9 days (2-16, SD 3.0) prior to injury. The median interval between the time of injury and initial WBCT assessment was 11.5 days (7-19, SD 3.2). For our total study population (n = 38), in healthy feet only, the median ILL distance measured 3.8 mm (2.2-5.4, SD 0.7) and the median DLL distance measured 0.9 mm (0.3-2.0, SD 0.3). Youden index identified the optimal threshold value at ≥2.7 mm (95% CI, 2.4-3.0). Eight of 38 patients (21%) were classified as having unstable injuries, with a median difference score of 4.6 mm (95% CI, 3.8-5.8). Instability was confirmed perioperatively in all 8 patients through a fluoroscopic stress test. In contrast, the 30 patients considered stable showed a median difference score of 0.9 mm (95% CI, 0.6-1.1).

**Table 1.** Patient Demographics (n = 38).

Mean age (SD)	36 (12.4)
Gender (male/female)	17 / 21
Side (left/right)	19 / 19
Mean BMI (SD)	25.4 (4.5)
Smoking (yes/no)	4 / 34
Sport-related injury (yes/no)	13 / 25
Plantar ecchymosis on initial contact (yes/no)	19 / 19
Initial NWB radiograph (negative/positive^a^)	20 / 18

Abbreviation: BMI, body mass index; NWB, nonweightbearing.

a Fractures detected by radiologist on initial evaluation.

**Table 2.** Fracture Patterns Observed on Nonweightbearing Computed Tomography Prior to Inclusion (n = 38).

	Medial Column	Middle Column	Lateral Column
No fracture present	19	0	21
Avulsion fracture	17	28	8
Intraarticular fracture	3	18	9
Extra-articular fracture	1	3	1

Of the 30 stable patients, 29 were reassessed after approximately 12 weeks, with a median follow-up time of 87 days (70-105, SD 8.7). One patient declined the WBCT at the scheduled follow-up because of an absence of symptoms. Instead, he was examined with bilateral weightbearing radiography, which showed no signs of side-by-side C1-M2 diastasis. Consequently, his primary WBCT images were included for ICC assessment, but he was excluded from the comparative imaging analyses. Among the remaining stable patients, the median difference score at 12-week follow-up was 0.6 mm (95% CI, 0.4-0.7), reflecting a statistically significant reduction (*P* = .0116) compared with the initial assessment.

**Figure 5.**
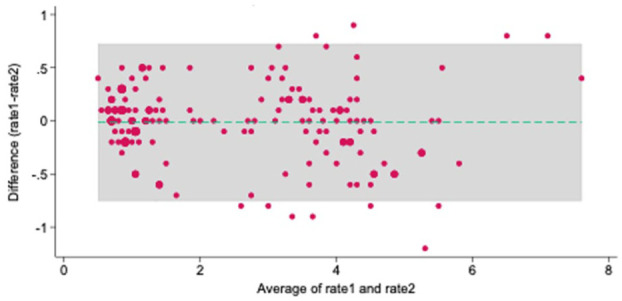
Bland Altman plot expressing limit of agreement for intraobserver agreement. 11 of 152 = 7.24% outside the limit of agreement; mean difference: −0.014; 95% limit of agreement (−0.753, 0.724); averages lie between 0.5 and 7.5.

In total, 138 individual WBCT images were assessed with our measurement protocol. When tested for agreement, we found an overall ICC: interrater agreement of 0.95. (95% CI, 0.93-0.96) for our 3 observers, and an intrarater agreement of 0.97 (95% CI, 0.96-0.98) (Figure 5).

## Discussion

In this prospective cohort of patients with subtle Lisfranc injuries, a >3 mm C1-M2 difference on WBCT under load identified all cases confirmed as unstable by intraoperative stress testing. This study supports WBCT as a reproducible, physiologically relevant tool for early detection of instability, potentially reducing delays to surgical management.

First, the preferred application and optimal timing is addressed. A common approach to investigate midfoot injuries is through bipedal weightbearing imaging.^4,25^ However, this method may allow for offloading of the injured foot, which can lead to an overestimation of Lisfranc stability. We recommend monopodial assessment to enhance the reliability of weightbearing scans. More importantly, allowing sufficient time for pain management, which enables patients to fully weightbear in a single stance, ensures adequate weight distribution to be applied on the injured foot. In a study involving 96 patients, Stødle et al^27^ reported Lisfranc joint assessments through either a stress-test or weightbearing radiographs conducted after 7-14 days. However, to our knowledge, there are no studies examining the optimal time frame for WBCT in Lisfranc diagnostics. Based on our findings, adequate interval from the date of injury to a fully weightbearing assessment was approximately 9 days. We also recommend that patients be allowed to bear weight as tolerated in a walking boot prior to the initial examination to help prepare them for the WBCT scans.

Furthermore, we investigate the measurement technique for Lisfranc joint stability. Currently, weightbearing conventional radiography is generally the preferred method to evaluate Lisfranc injuries. However, obtaining consistent and reproducible radiographic images can be challenging because of 2-dimensional bone superimposition and the necessity of craniocaudal X-ray beam projection to effectively visualize midfoot pathology.^16^ In contrast, CT overcomes this limitation by allowing reconstruction in multiple planes, increasing the diagnostic accuracy, which is especially important for subtle Lisfranc injuries.^3,24^ Despite this, no studies have been published that directly compare weightbearing radiography with WBCT for Lisfranc assessment. Instead, research have focused on validating various CT measurement protocols. Sripanich et al^25^ were the first to present a specific technique to measure the ILL distance with WBCT. This retrospective study analyzed 96 WBCT scans of healthy feet and introduced 2 similar methods for locating a reference point for the ligament: one involved calculating an exact distance at the base of M2, whereas the other relied on bony landmarks for distance approximation. Although intrarater agreement was reported to be excellent for both approaches, we encountered challenges in replicating both methods before study start because of a lack of coordination among the 3 orthogonal planes. Measurement accuracy is vital, as misalignment can significantly impact the overall evaluation. This is especially true for the second metatarsal, whose triangular shape in the coronal plane causes the distance to the medial cuneiform to vary depending on whether measurements are taken dorsally or plantarly. Falcon et al^4^ conducted a comparable retrospective study using WBCT to evaluate normal and pathological conditions of the Lisfranc ligament in 56 patients. They highlighted WBCT's comparative capability and the diagnostic potential of C1-M2 measurements, reporting a median C1-M2 distance of 3.7 mm in healthy feet, close to our finding of 3.8 mm. However, the method's reliance on distance measurements using axial slices at optimal articulation, as determined by a musculoskeletal radiologist, limits its practical application. The first publications to explore a different approach than a 1-dimensional, C1-M2 measurement, was Bhimani et al^2^ who evaluated additional sensitivity of 2-dimensional area and 3-dimensional volume measurements of the Lisfranc joint complex with WBCT. Fourteen patients with surgically confirmed Lisfranc injuries were retrospectively assessed, and they found coronal measurements to be more sensitive than axial based on the dorsolateral direction of interarticular diastasis. Consequently, this radiologic plane was preferred for conclusive measurements in our protocol. When comparing techniques, the volumetric method showed the highest sensitivity and specificity to detect subtle instability patterns. This is not surprising, since the anticipated widening of the injured C1-M2 Lisfranc interval represents an expanding, 3-dimensional space. Although accurate, volumetric measurement techniques are still regarded as a novel method and are seldom used in clinical practice. We propose that a combination of measurements—including the DLL and ILL distances—allows for the assessment of 2 of the vital structures necessary for maintaining Lisfranc joint stability. In the present study, we have validated a triplane protocol that uses anatomical landmarks to measure joint diastasis, making it consistent and replicable in clinical practice without the need for advanced calculations, or volume detection. Conversely, perhaps in the future, deep learning methods or artificial intelligence solutions may prove to be more effective tools for evaluating subtle volumetric changes in the Lisfranc joint complex.^1^

Accurately distinguishing between stable and unstable Lisfranc injuries is critical for improving patient outcomes.^9,15^ For this, the key questions remain: Can an absolute threshold for Lisfranc joint stability be established, and how much confidence can we place in our initial assessments? Sripanich et al^23^ published a cadaver study that investigated the stability threshold of the Lisfranc complex after sequential sectioning of the Lisfranc ligaments. They proposed that a 1.5-mm increase in the C1-M2 difference indicates a complete ligament rupture. However, the clinical applicability of these findings is limited, as Lisfranc injuries are often heterogeneous and isolated Lisfranc ligament injuries are rare. Nevertheless, in our study, we established a difference score threshold of 3.0 mm by combining both the DLL and ILL a priori. Notably, our retrospective analysis suggests that this threshold is closely aligned with the optimal value of 2.7 mm derived from the Youden index. Biomechanically, among the 3 components of the Lisfranc ligament complex, the interosseous portion exhibits the most robust biomechanical properties and thereby is considered to play the most important role in maintaining Lisfranc joint stability.^22^ An avulsion fracture in this area, commonly referred to as a “fleck sign,” is a strong indicator of a Lisfranc injury, leading to the assumption that ruptures in this area signify instability.^14^ However, as demonstrated in this study, the presence of an interosseous fleck sign does not necessarily indicate an unstable injury. The actual measurement application in patients with the presence of an avulsion fracture inside the C1-M2 interval is yet another issue that has not been addressed in published literature. Yet, the most important strength of this study lies in its clinical approach and prospective assessment methodology, being the first study to publish follow-up results after an initial WBCT assessment. All our classified unstable injuries showed substantial signs of joint instability during perioperative stress-test. Equally, persistent stability was verified at scheduled follow-up with the injuries classified as stable. Additionally, with our rehabilitation protocol, which includes weightbearing in a walking boot as tolerated after WBCT assessment, we observed a significant reduction in the C1-M2 difference score among our patients, with no individuals showing signs of displacement above our established threshold for joint instability.

We acknowledge the limitations to our research. Firstly, the study was not powered statistically prior to inclusion but rather reflects the outcomes of a consecutive cohort over a period of 13 months. Consequently, this limits precision when defining stability thresholds and the results should be taken with some precaution. Moreover, to validate our clinical findings of instability, we performed a fluoroscopic stress-test under general anesthesia. We recognize that this method is subjective, can vary between examiners, and may overestimate instability. Additionally, for obvious reasons, we did not sedate all patients to conduct identical comparative examinations for those with stable Lisfranc injuries. We recognize that this introduces verification bias into our results. However, to compensate for this, all stable patients were reevaluated with follow-up WBCT for stability confirmation. Furthermore, this study focuses on stability evaluation, meaning we have deliberately not included PROMs evaluations to the results. Still, it is important to recognize that stability is not the only important factor to consider when assessing an acute Lisfranc injury. In our practice, patients with either comminuted fractures or multiple intraarticular fracture lines, especially in TMT 2, should be evaluated for primary arthrodesis, even in the absence of C1-M2 diastasis on WBCT.

## Conclusion

This article presents the first prospective study using bilateral WBCT to assess nondisplaced, subtle Lisfranc injuries. The distance of the dorsal Lisfranc ligament and the interosseous Lisfranc ligament were measured when the patient was able to fully bear weight, with a combined, side-to-side difference score of 3 mm serving as a reliable indicator of stability. Furthermore, our triplane, anatomically based measuring approach demonstrated excellent reliability.

## Supplemental Material

sj-pdf-1-fai-10.1177_10711007251374674 – Supplemental material for Prospective Validation of a Weightbearing CT Stability Threshold for Subtle Lisfranc Injuries Supplemental material, sj-pdf-1-fai-10.1177_10711007251374674 for Prospective Validation of a Weightbearing CT Stability Threshold for Subtle Lisfranc Injuries by Magnus Poulsen, Stephan M. Röhrl, Anselm Schulz and Are H. Stødle in Foot & Ankle International
